# Fair Refinement for Asynchronous Session Types

**DOI:** 10.1007/978-3-030-71995-1_8

**Published:** 2021-03-23

**Authors:** Mario Bravetti, Julien Lange, Gianluigi Zavattaro

**Affiliations:** 8 University of Oxford, Oxford, UK; 9 Sorbonne Université - LIP6, Paris, France; 10 University of Bologna / INRIA FoCUS Team, Bologna, Italy; 11 Royal Holloway, University of London, Egham, UK

**Keywords:** Session types, Asynchronous communication, Subtyping

## Abstract

Session types are widely used as abstractions of asynchronous message passing systems. Refinement for such abstractions is crucial as it allows improvements of a given component without compromising its compatibility with the rest of the system. In the context of session types, the most general notion of refinement is the asynchronous session subtyping, which allows to anticipate message emissions but only under certain conditions. In particular, asynchronous session subtyping rules out candidates subtypes that occur naturally in communication protocols where, e.g., two parties simultaneously send each other a finite but unspecified amount of messages before removing them from their respective buffers. To address this shortcoming, we study fair compliance over asynchronous session types and fair refinement as the relation that preserves it. This allows us to propose a novel variant of session subtyping that leverages the notion of controllability from service contract theory and that is a sound characterisation of fair refinement. In addition, we show that both fair refinement and our novel subtyping are undecidable. We also present a sound algorithm, and its implementation, which deals with examples that feature potentially unbounded buffering.

## 1 Introduction

The coordination of software components via message-passing techniques is becoming increasingly popular in modern programming languages and development methodologies based on actors and microservices, e.g., Rust, Go, and the Twelve-Factor App methodology [1]. Often the communication between two concurrent or distributed components takes place over point-to-point FIFO channels.

Abstract models such as communicating finite-state machines [5] and asynchronous session types [21] are essential to reason about the correctness of such systems in a rigorous way. In particular these models are important to reason about mathematically grounded techniques to improve concurrent and distributed systems in a compositional way. The key question is whether a component can be *refined* independently of the others, without compromising the

* Research partly supported by the H2020-MSCA-RISE project ID 778233 "Behavioural Application Program Interfaces (BEHAPI)".

correctness of the whole system. In the theory of session types, the most general notion of refinement is the asynchronous session subtyping [14, 15, 26], which leverages asynchrony by allowing the refined component to anticipate message emissions, but only under certain conditions. Notably asynchronous session subtyping rules out candidate subtypes that occur naturally in communication protocols where, e.g., two parties simultaneously send each other a finite but unspecified amount of messages before removing them from their buffers.

We illustrate this key limitation of asynchronous session subtyping with Figure 1, which depicts possible communication protocols between a spacecraft and a ground station. For convenience, the protocols are represented as session types (bottom) and equivalent communicating finite-state machines (top). Consider $T_S$ and $T_G$ first. Session type $T_S$ is the abstraction of the spacecraft. It may send a finite but unspecified number of telemetries ($tm$), followed by a message *over* — this phase of the protocol typically models a `for` loop and its exit. In the second phase, the spacecraft receives a number of telecommands ($tc$), followed by a message *done*. Session type $T_G$ is the abstraction of the ground station. It is the *dual* of $T_S$, written $\overline{T_S}$, as required in standard binary session types without subtyping. Since $T_G$ and $T_S$ are dual of each other, the theory of session types guarantees that they form a *correct composition*, namely both parties terminate successfully, with empty queues.

However, it is clear that this protocol is not efficient: the communication is half-duplex, i.e., it is never the case that more than one party is sending at any given time. Using full-duplex communication is crucial in distributed systems with intermittent connectivity, e.g., in this case ground stations are not always visible from low orbit satellites.

The abstraction of a more efficient ground station is given by type $T'_G$, which sends telecommands before receiving telemetries. It is clear that $T'_G$ and $T_S$ forms a correct composition. Unfortunately $T'_G$ is not an asynchronous subtype of $T_G$ according to earlier definitions of session subtyping [14,15,26]. Hence they cannot formally guarantee that $T'_G$ is a safe replacement for $T_G$. Concretely, these subtyping relations allow for anticipation of emissions (output) only when they are preceded by a *bounded* number of receptions (input), but this does not hold between $T'_G$ and $T_G$ because the latter starts with a loop of inputs. Note that the composition of $T'_G$ and $T_S$ is not existentially bounded, hence it cannot be verified by related communicating finite-state machines techniques [4,19,20,24].

In this paper we address this limitation of previous asynchronous session subtyping relations. To do this, we move to an alternative notion of correct composition. In [14] the authors show that their subtyping relation is fully abstract w.r.t. the notion of *orphan-message-free* composition. More precisely, it captures exactly a notion of refinement that preserves the possibility for all sent messages to be consumed along *all* possible computations of the receiver. In the spacecraft example, given the initial loop of outputs in $T'_G$, there is an extreme case in which it performs infinitely many outputs without consuming any incoming messages. Nevertheless, this limit case cannot occur under the natural assumption that

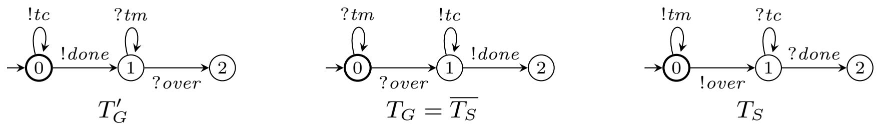

$$T'_G = \mu\mathbf{t}. \oplus \{tc : \mathbf{t}, done : \mu\mathbf{t}'. \&\{tm : \mathbf{t}', over : \mathbf{end}\}\}$$
$$T_G = \mu\mathbf{t}. \&\{tm : \mathbf{t}, over : \mu\mathbf{t}'. \oplus \{tc : \mathbf{t}', done : \mathbf{end}\}\}$$
$$T_S = \mu\mathbf{t}. \oplus \{tm : \mathbf{t}, over : \mu\mathbf{t}'. \&\{tc : \mathbf{t}', done : \mathbf{end}\}\}$$

**Fig. 1.** Satellite protocols. $T'_G$ is the refined session type of the ground station, $T_G$ is the session type of ground station, and $T_S$ is the session type of the spacecraft.

the loop of outputs eventually terminates, i.e., only a finite (but unspecified) amount of messages can be emitted.

The notion of correct composition that we use is based on *fair* compliance, which requires each component to always be able to eventually reach a successful final state. This is a liveness property, holding under *full fairness* [32], used also in the theory of should testing [30] where "every reachable state is required to be on a path to success". This is a natural constraint since even programs that conceptually run indefinitely must account for graceful termination (e.g., to release acquired resources). Previously, fair compliance has been considered to reason formally about component/service composition with *synchronous* session types [29] and *synchronous* behavioural contracts [11]. A preliminary formalisation of fair compliance for *asynchronous* behavioural contracts was presented in [10], but considering an operational model very different from session types.

Given a notion of fair compliance defined on an operational model for asynchronous session types, we define *fair refinement* as the relation that preserves it. Then, we propose a novel variant of session subtyping called *fair asynchronous session subtyping*, that leverages the notion of controllability from service contract theory, and which is a sound characterisation of fair refinement. We show that both fair refinement and fair asynchronous session subtyping are undecidable, but give a sound algorithm for the latter. Our algorithm covers session types that exhibit complex behaviours (including the spacecraft example and variants). Our algorithm has been implemented in a tool available online [31].

*Structure of the paper* The rest of this paper is structured as follows. In § 2 we recall syntax and semantics of asynchronous session types, we define *fair compliance* and the corresponding *fair refinement*. In § 3 we introduce *fair asynchronous subtyping*, the first relation of its kind to deal with examples such as those in Figure 1. In § 4 we propose a sound algorithm for subtyping that supports examples with unbounded accumulations, including the ones discussed in this paper. In § 5 we discuss the implementation of this algorithm. Finally, in § 6 we discuss related works and future work. We give proofs for all our results and examples of output from our tool in [9].

## 2 Refinement for Asynchronous Session Types

In this section we first recall the syntax of two-party session types, their reduction semantics, and a notion of compliance centred on the successful termination of interactions. We define our notion of refinement based on this compliance and show that it is generally undecidable whether a type is a refinement of another.

### 2.1 Preliminaries: Asynchronous Session Types

*Syntax* The formal syntax of two-party session types is given below. We follow the simplified notation used in, e.g., [7,8], without dedicated constructs for sending an output/receiving an input. Additionally we abstract away from message payloads since they are orthogonal to the results of this paper.

**Definition 1 (Session Types).** *Given a set of labels $\mathcal{L}$, ranged over by $l$, the syntax of two-party session types is given by the following grammar:*

$$T \;::=\; \oplus\{l_i : T_i\}_{i\in I} \quad|\quad \&\{l_i : T_i\}_{i\in I} \quad|\quad \mu\mathbf{t}.T \quad|\quad \mathbf{t} \quad|\quad \mathbf{end}$$

Output selection $\oplus\{l_i : T_i\}_{i\in I}$ represents a guarded internal choice, specifying that a label $l_i$ is sent over a channel, then continuation $T_i$ is executed. Input branching $\&\{l_i : T_i\}_{i\in I}$ represents a guarded external choice, specifying a protocol that waits for messages. If message $l_i$ is received, continuation $T_i$ takes place. In selections and branchings each branch is tagged by a label $l_i$, taken from a global set of labels $\mathcal{L}$. In each selection/branching, these labels are assumed to be pairwise distinct. In the sequel, we leave implicit the index set $i \in I$ in input branchings and output selections when it is clear from the context. Types $\mu\mathbf{t}.T$ and $\mathbf{t}$ denote standard recursion constructs. We assume recursion to be guarded in session types, i.e., in $\mu\mathbf{t}.T$, the recursion variable $\mathbf{t}$ occurs within the scope of a selection or branching. Session types are closed, i.e., all recursion variables $\mathbf{t}$ occur under the scope of a corresponding binder $\mu\mathbf{t}.T$. Terms of the session syntax that are not closed are dubbed (session) terms. Type **end** denotes the end of the interactions.

The dual of session type $T$, written $\overline{T}$, is inductively defined as follows: $\overline{\oplus\{l_i : T_i\}_{i\in I}} = \&\{l_i : \overline{T}_i\}_{i\in I}$, $\overline{\&\{l_i : T_i\}_{i\in I}} = \oplus\{l_i : \overline{T}_i\}_{i\in I}$, $\overline{\mathbf{end}} = \mathbf{end}$, $\overline{\mathbf{t}} = \mathbf{t}$, and $\overline{\mu\mathbf{t}.T} = \mu\mathbf{t}.\overline{T}$.

*Operational characterisation* Hereafter, we let $\omega$ range over words in $\mathcal{L}^*$, write $\epsilon$ for the empty word, and write $\omega_1 \cdot \omega_2$ for the concatenation of words $\omega_1$ and $\omega_2$, where each word may contain zero or more labels. Also, we write $T\{{}^{T'}\!/\mathbf{t}\}$ for $T$ where every free occurrence of $\mathbf{t}$ is replaced by $T'$.

We give an asynchronous semantics of session types via transition systems whose states are configurations of the form: $[T_1, \omega_1]|[T_2, \omega_2]$ where $T_1$ and $T_2$ are session types equipped with two sequences $\omega_1$ and $\omega_2$ of incoming messages (representing unbounded buffers). We use $s$, $s'$, etc. to range over configurations.

In this paper, we use explicit unfoldings of session types, as defined below.

**Definition 2 (Unfolding).** *Given session type $T$, we define* $\mathsf{unfold}(T)$*:*

$$\mathsf{unfold}(T) = \begin{cases} \mathsf{unfold}(T'\{^T/_\mathbf{t}\}) & \text{if } T = \mu\mathbf{t}.T' \\ T & \text{otherwise} \end{cases}$$

Definition 2 is standard, e.g., an equivalent function is used in the first session subtyping [18]. Notice that $\mathsf{unfold}(T)$ unfolds all the recursive definitions in front of $T$, and it is well defined for session types with guarded recursion.

**Definition 3 (Transition Relation).** *The transition relation $\rightarrow$ over configurations is the minimal relation satisfying the rules below (plus symmetric ones):*

1. *if $j \in I$ then $[\oplus\{l_i : T_i\}_{i\in I}, \omega_1]|[T_2, \omega_2] \rightarrow [T_j, \omega_1]|[T_2, \omega_2 \cdot l_j]$;*
2. *if $j \in I$ then $[\&\{l_i : T_i\}_{i\in I}, l_j \cdot \omega_1]|[T_2, \omega_2] \rightarrow [T_j, \omega_1]|[T_2, \omega_2]$;*
3. *if $[\mathsf{unfold}(T_1), \omega_1]|[T_2, \omega_2] \rightarrow s$ then $[T_1, \omega_1]|[T_2, \omega_2] \rightarrow s$.*

*We write $\rightarrow^*$ for the reflexive and transitive closure of the $\rightarrow$ relation.*

Intuitively a configuration $s$ reduces to configuration $s'$ when either (1) a type outputs a message $l_j$, which is added at the end of its partner's queue; (2) a type consumes an expected message $l_j$ from the head of its queue; or (3) the unfolding of a type can execute one of the transitions above.

Next, we define successful configurations as those configurations where both types have terminated (reaching **end**) and both queues are empty. We use this to give our definition of compliance which holds when it is possible to reach a successful configuration from all reachable configurations.

**Definition 4 (Successful Configuration).** *The notion of successful configuration is formalised by a predicate $s\surd$ defined as follows:*

$$[T, \omega_T]|[S, \omega_S]\surd \quad \text{iff} \quad \mathsf{unfold}(T) = \mathsf{unfold}(S) = \mathbf{end} \quad \text{and} \quad \omega_T = \omega_S = \epsilon$$

**Definition 5 (Compliance).** *Given a configuration $s$ we say that it is a correct composition if, whenever $s \rightarrow^* s'$, there exists a configuration $s''$ such that $s' \rightarrow^* s''$ and $s''\surd$.*
*Two session types $T$ and $S$ are* compliant *if $[T, \epsilon]|[S, \epsilon]$ is a correct composition.*

Observe that our definition of compliance is stronger than what is generally considered in the literature on session types, e.g., [16, 23, 24], where two types are deemed compliant if all messages that are sent are eventually received, and each non-terminated type can always eventually make a move. Compliance is analogous to the notion of *correct session* in [29] but in an asynchronous setting.

A consequence of Definition 5 is that it is generally *not* the case that a session type $T$ is compliant with its dual $\overline{T}$, as we show in the example below.

*Example 1.* The session type $T = \&\{l_1 : \mathbf{end},\ l_2 : \mu\mathbf{t}. \oplus \{l_3 : \mathbf{t}\}\}$ and its dual $\overline{T} = \oplus\{l_1 : \mathbf{end},\ l_2 : \mu\mathbf{t}.\&\{l_3 : \mathbf{t}\}\}$ are not compliant. Indeed, when $\overline{T}$ sends label $l_2$, the configuration $[\mathbf{end}, \epsilon]|[\mathbf{end}, \epsilon]$ is no longer reachable.

## 2.2 Fair Refinement for Asynchronous Session Types

We introduce a notion of refinement that preserves compliance. This follows previous work done in the context of behavioural contracts [11] and *synchronous* multi-party session types [29]. The key difference with these works is that we are considering asynchronous communication based on (unbounded) FIFO queues. Asynchrony makes fair refinement undecidable, as we show below.

**Definition 6 (Refinement).** *A session type $T$ refines $S$, written $T \sqsubseteq S$, if for every $S'$ s.t. $S$ and $S'$ are compliant then $T$ and $S'$ are also compliant.*

In contrast to traditional (synchronous and asynchronous) subtyping for session types [14, 18, 26], this refinement is not covariant on outputs, i.e., it does not always allow a refined type to have output selections with less labels.[3]

*Example 2.* Let $T = \mu\mathbf{t}. \oplus \{l_1 : \mathbf{t}\}$ and $S = \mu\mathbf{t}. \oplus \{l_1 : \mathbf{t},\ l_2 : \mathbf{end}\}$. We have that $T$ is a synchronous (and asynchronous) subtype of $S$. However $T$ is *not* a refinement of $S$. In particular, the type $\overline{S} = \mu\mathbf{t}. \&\{l_1 : \mathbf{t},\ l_2 : \mathbf{end}\}$ is compliant with $S$ but not with $T$, since $T$ does not terminate.

Next, we show that the refinement relation $\sqsubseteq$ is generally undecidable. The proof of undecidability exploits results from the tradition of computability theory, i.e., Turing completeness of queue machines. The crux of the proof is to reduce the problem of checking the reachability of a given state in a queue machine to the problem of checking the refinement between two session types.

*Preliminaries* Below we consider only state reachability in queue machines, and not the typical notion of the language recognised by a queue machine (see, e.g., [7] for a formalisation of queue machines). Hence, we use a simplified formalisation, where no input string is considered.

**Definition 7 (Queue Machine).** *A queue machine $M$ is defined by a six-tuple $(Q, \Sigma, \Gamma, \$, s, \delta)$ where:*

- *$Q$ is a finite set of states;*
- *$\Sigma \subset \Gamma$ is a finite set denoting the input alphabet;*
- *$\Gamma$ is a finite set denoting the queue alphabet (ranged over by $A, B, C, X$);*
- *$\$ \in \Gamma - \Sigma$ is the initial queue symbol;*
- *$s \in Q$ is the start state;*
- *$\delta : Q \times \Gamma \to Q \times \Gamma^*$ is the transition function ($\Gamma^*$ is the set of sequences of symbols in $\Gamma$).*

Considering a queue machine $M = (Q, \Sigma, \Gamma, \$, s, \delta)$, a *configuration* of $M$ is an ordered pair $(q, \gamma)$ where $q \in Q$ is its *current state* and $\gamma \in \Gamma^*$ is the *queue*. The starting configuration is $(s, \$)$, composed of the start state $s$ and the initial queue symbol $\$$.

Next, we define the transition relation ($\to_M$), leading a configuration to another, and the related notion of state reachability.

[3] The synchronous subtyping in [18] follows a channel-oriented approach; hence it has the opposite direction and is contravariant on outputs.

**Definition 8 (State Reachability).** *Given a machine* $M = (Q, \Sigma, \Gamma, \$, s, \delta)$*, the transition relation* $\rightarrow_M$ *over configurations* $Q \times \Gamma^*$ *is defined as follows. For* $p, q \in Q$*,* $A \in \Gamma$*, and* $\alpha, \gamma \in \Gamma^*$*, we have* $(p, A\alpha) \rightarrow_M (q, \alpha\gamma)$ *whenever* $\delta(p, A) = (q, \gamma)$*. Let* $\rightarrow_M^*$ *be the reflexive and transitive closure of* $\rightarrow_M$*.*
*A target state* $q_f \in Q$ *is* reachable *in* $M$ *if there is* $\gamma \in \Gamma^*$ *s.t.* $(s, \$) \rightarrow_M^* (q_f, \gamma)$*.*

Since queue machines can deterministically encode Turing machines (see, e.g., [7]), checking state reachability for queue machines is undecidable.

**Theorem 1.** *Given a queue machine* $M$ *and a target state* $q_f$ *it is possible to reduce the problem of checking the reachability of* $q_f$ *in* $M$ *to the problem of checking refinement between two session types.*

In the light of the undecidability of reachability in queue machines, we can conclude that refinement (Definition 6) is also undecidable.

### 2.3 Controllability for Asynchronous Session Types

Given a notion of compliance, controllability amounts to checking the existence of a compliant partner (see, e.g., [12, 25, 33]). In our setting, a session type is *controllable* if there exists another session type with which it is compliant.

Checking for controllability algorithmically is not trivial as it requires to consider infinitely many potential partners. For the synchronous case, an algorithmic characterisation was studied in [29]. In the asynchronous case, the problem is even harder because each of the infinitely many potential partners may generate an infinite state computation (due to unbounded buffers). The main contribution of this subsection is to give an algorithmic characterisation of controllability in the asynchronous setting. Doing this is important because controllability is an essential ingredient for defining fair asynchronous subtyping, see Section 3.

**Definition 9 (Characterisation of Controllability,** $T\,\mathsf{ctrl}$**).** *Given a session type* $T$*, we define the judgement* $T\,\mathsf{ok}$ *inductively as follows:*

$$\frac{}{\mathbf{end}\,\mathsf{ok}} \qquad \frac{\mathbf{end} \in T \qquad T\{\mathbf{end}/\mathbf{t}\}\,\mathsf{ok}}{\mu\mathbf{t}.T\,\mathsf{ok}} \qquad \frac{T\,\mathsf{ok}}{\&\{l : T\}\,\mathsf{ok}} \qquad \frac{\forall i \in I.\ T_i\,\mathsf{ok}}{\oplus\{l_i : T_i\}_{i\in I}\,\mathsf{ok}}$$

*where* $\mathbf{end} \in T$ *holds if* $\mathbf{end}$ *occurs in* $T$*.*

*We write* $T\,\mathsf{ctrl}$ *if there exists* $T'$ *such that (i)* $T'$ *is obtained from* $T$ *by syntactically replacing every input prefix* $\&\{l_i : T_i\}_{i\in I}$ *occurring in* $T$ *with a term* $\&\{l_j : T_j\}$ *(with* $j \in I$*) and (ii)* $T'\,\mathsf{ok}$ *holds.*

Notice that a type $T$ such that $T\,\mathsf{ctrl}$ is indeed controllable, in that $\overline{T'}$, the dual of type $T'$ considered above, is compliant with $T$ (the predicate $\mathbf{end} \in T$ in the premise of the rule for recursion guarantees that a successful configuration is always reachable while looping). Moreover the above definition naturally yields a simple algorithm that decides whether or not $T\,\mathsf{ctrl}$ holds for a type $T$, i.e., we first pick a single branch for each input prefix syntactically occurring in $T$ (there are finitely many of them) and then we inductively check if $T'\,\mathsf{ok}$ holds.

The following theorem shows that the judgement $T\,\mathsf{ctrl}$, as defined above, precisely characterises controllability (i.e., the existence of a compliant type).

**Theorem 2.** *$T$ $\mathsf{ctrl}$ holds if and only if there exists a session type $S$ such that $T$ and $S$ are compliant.*

*Example 3.* Consider the session type $T = \mu\mathbf{t}.\ \&\{l_1 : \&\{l_2 : \oplus\{l_4 : \mathbf{end},\ l_5 : \mu\mathbf{t}'.\oplus\{l_6 : \mathbf{t}'\}\},\ l_3 : \mathbf{t}\}\}$. $T$ $\mathsf{ctrl}$ does *not* hold because it is not possible to construct a $T'$ as specified in Definition 9 for which $T'$ $\mathsf{ok}$ holds. By Theorem 2, there is no session type $S$ that is compliant with $T$. Hence $T$ is not controllable.

## 3 Fair Asynchronous Session Subtyping

In this section, we present our novel variant of asynchronous subtyping which we dub *fair asynchronous subtyping.*

We need to define a distinctive notion of unfolding. Function $\mathsf{selUnfold}(T)$ unfolds type $T$ by replacing recursion variables with their corresponding definitions only if they are guarded by an output selection. In the definition, we use the predicate $\oplus g(\mathbf{t}, T)$ which holds if all instances of variable $\mathbf{t}$ are output selection guarded, i.e., $\mathbf{t}$ occurs free in $T$ only inside subterms $\oplus\{l_i : T_i\}_{i\in I}$.

**Definition 10 (Selective Unfolding).** *Given a term $T$, define* $\mathsf{selUnfold}(T) =$

$$\begin{cases} \oplus\{l_i : T_i\}_{i\in I} & \text{if } T = \oplus\{l_i : T_i\}_{i\in I} \\ \&\{l_i : \mathsf{selUnfold}(T_i)\}_{i\in I} & \text{if } T = \&\{l_i : T_i\}_{i\in I} \\ T'\{^{\mu\mathbf{t}.T'}/_{\mathbf{t}}\} & \text{if } T = \mu\mathbf{t}.T',\ \oplus g(\mathbf{t}, T') \\ \mu\mathbf{t}.\mathsf{selUnfold}(\mathsf{selRepl}(\mathbf{t}, \hat{\mathbf{t}}, T')\{^{\mu\mathbf{t}.T'}/_{\hat{\mathbf{t}}}\}) \text{ with } \hat{\mathbf{t}} \text{ fresh} & \text{if } T = \mu\mathbf{t}.T',\ \neg\oplus g(\mathbf{t}, T') \\ \mathbf{t} & \text{if } T = \mathbf{t} \\ \mathbf{end} & \text{if } T = \mathbf{end} \end{cases}$$

*where,* $\mathsf{selRepl}(\mathbf{t}, \hat{\mathbf{t}}, T')$ *is obtained from $T'$ by replacing the free occurrences of* $\mathbf{t}$ *that are inside a subterm $\oplus\{l_i : S_i\}_{i\in I}$ of $T'$ by $\hat{\mathbf{t}}$.*

*Example 4.* Consider the type $T = \mu\mathbf{t}.\&\{l_1 : \mathbf{t},\ l_2 : \oplus\{l_3 : \mathbf{t}\}\}$, then we have

$$\mathsf{selUnfold}(T) = \mu\mathbf{t}.\&\{l_1 : \mathbf{t},\ l_2 : \oplus\{l_3 : \mu\mathbf{t}.\ \&\{l_1 : \mathbf{t},\ l_2 : \oplus\{l_3 : \mathbf{t}\}\}\}\}$$

i.e., the type is only unfolded within output selection sub-terms. Note that $\hat{\mathbf{t}}$ is used to identify where unfolding must take place, e.g.,
$\mathsf{selRepl}(\mathbf{t}, \hat{\mathbf{t}}, \&\{l_1 : \mathbf{t},\ l_2 : \oplus\{l_3 : \mathbf{t}\}\}) = \&\{l_1 : \mathbf{t},\ l_2 : \oplus\{l_3 : \hat{\mathbf{t}}\}\}$.

The last auxiliary notation required to define our notion of subtyping is that of *input contexts*, which are used to record inputs that may be delayed in a candidate super-type.

**Definition 11 (Input Context).** *An input context $\mathcal{A}$ is a session type with several holes defined by the syntax:*

$$\mathcal{A} \ ::= \quad [\,]^k \quad | \quad \&\{l_i : \mathcal{A}_i\}_{i\in I} \quad | \quad \mu\mathbf{t}.\mathcal{A} \quad | \quad \mathbf{t}$$

*where the holes* $[\,]^k$*, with* $k \in K$*, of an input context* $\mathcal{A}$ *are assumed to be pairwise distinct. We assume that recursion is guarded, i.e., in an input context* $\mu\mathbf{t}.\mathcal{A}$*, the recursion variable* $\mathbf{t}$ *must occur within a subterm* $\&\{l_i : \mathcal{A}_i\}_{i\in I}$*.*

*We write holes*$(\mathcal{A})$ *for the set of hole indices in* $\mathcal{A}$*. Given a type* $T_k$ *for each* $k \in K$*, we write* $\mathcal{A}[T_k]^{k\in K}$ *for the type obtained by filling each hole* $k$ *in* $\mathcal{A}$ *with the corresponding* $T_k$*.*

In contrast to previous work [6,7,13–15,26], these input contexts may contain recursive constructs. This is crucial to deal with examples such as Figure 1.

We are now ready to define the *fair asynchronous subtyping* relation, written $\leq$. The rationale behind asynchronous session subtyping is that under asynchronous communication it is unobservable whether or not an output is anticipated before an input, as long as this output is executed along all branches of the candidate super-type. Besides the usage of our new recursive input contexts the definition of fair asynchronous subtyping differs from those in [6,7,13–15,26] in that controllability plays a fundamental role: the subtype is not required to mimic supertype inputs leading to uncontrollable behaviours.

**Definition 12 (Fair Asynchronous Subtyping,** $\leq$**).**
*A relation* $\mathcal{R}$ *on session types is a controllable subtyping relation whenever* $(T, S) \in \mathcal{R}$ *implies:*

1. *if* $T = \mathbf{end}$ *then* $\mathsf{unfold}(S) = \mathbf{end}$*;*
2. *if* $T = \mu\mathbf{t}.T'$ *then* $(T'\{T/\mathbf{t}\}, S) \in \mathcal{R}$*;*
3. *if* $T = \&\{l_i : T_i\}_{i\in I}$ *then* $\mathsf{unfold}(S) = \&\{l_j : S_j\}_{j\in J}$*,* $I \supseteq K$*, and* $\forall k \in K.\,(T_k, S_k) \in \mathcal{R}$*, where* $K = \{k \in J \mid S_k$ *is controllable*$\}$*;*
4. *if* $T = \oplus\{l_i : T_i\}_{i\in I}$ *then* $\mathsf{selUnfold}(S) = \mathcal{A}[\oplus\{l_i : S_{ki}\}_{i\in I}]^{k\in K}$ *and* $\forall i \in I.\,(T_i, \mathcal{A}[S_{ki}]^{k\in K}) \in \mathcal{R}$*.*

$T$ *is a controllable subtype of* $S$ *if there is a controllable subtyping relation* $\mathcal{R}$ *s.t.* $(T, S) \in \mathcal{R}$*.*
$T$ *is a* fair asynchronous subtype *of* $S$*, written* $T \leq S$*, whenever:* $S$ *controllable implies that* $T$ *is a controllable subtype of* $S$*.*

Notice that the top-level check for controllability in the above definition is consistent with the inner controllability checks performed in Case (3).

*Subtyping simulation game* Session type $T$ is a fair asynchronous subtype of $S$ if $S$ is not controllable or if $T$ is a controllable subtype of $S$. Intuitively, the above co-inductive definition says that it is possible to play a simulation game between a subtype $T$ and its supertype $S$ as follows. Case (1) says that if $T$ is the **end** type, then $S$ must also be **end**. Case (2) says that if $T$ is a recursive definition, then it simply unfolds this definition while $S$ does not need to reply. Case (3) says that if $T$ is an input branching, then the sub-terms in $S$ that are controllable can reply by inputting at most some of the labels $l_i$ in the branching (contravariance of inputs), and the simulation game continues (see Example 5). Case (4) says that if $T$ is an output selection, then $S$ can reply by outputting *all* the labels $l_i$ in the selection, possibly after executing some inputs, after which the simulation game continues. We comment further on Case (4) with Example 6.

*Example 5.* Consider $T = \&\{l_1 : \mathbf{end},\ l_2 : \mathbf{end}\}$ and $S = \&\{l_1 : \mathbf{end},\ l_3 : \mu\mathbf{t}.\oplus\{l_4 : \mathbf{t}\}\}$. We have $T \leq S$. Once branch $l_3$, that is uncontrollable, is removed from $S$, we can apply contravariance for input branching. We have $I = \{1, 2\} \supseteq \{1\} = K$ in Definition 12.

*Example 6.* Consider $T_G$ and $T'_G$ from Figure 1. For the pair $(T'_G, T_G)$, we apply Case (4) of Definition 12 for which we compute

$$\mathsf{selUnfold}(T_G) = \mathcal{A}[\oplus\{tc : \mu\mathbf{t}'. \oplus \{tc : \mathbf{t}', done : \mathbf{end}\}, done : \mathbf{end}\}]$$

with $\mathcal{A} = \mu\mathbf{t}.\&\{tm : \mathbf{t}, over : [\,]^1\}$. Observe that $\mathcal{A}$ contains a recursive sub-term, such contexts are not allowed in previous works [14, 15, 26].

The use of selective unfolding makes it possible to express $T_G$ in terms of a *recursive* input context $\mathcal{A}$ with holes filled by types (i.e., closed terms) that start with an output prefix. Indeed selective unfolding does not unfold the recursion variable $\mathbf{t}$ (*not* guarded by an output selection), which becomes part of the input context $\mathcal{A}$. Instead it unfolds the recursion variable $\mathbf{t}'$ (which is guarded by an output selection) so that the term that fills the hole, which is required to start with an output prefix, is a closed term.

Case (4) of Definition 12 requires us to check that the following pairs are in the relation: (*i*) $(T'_G, \mathcal{A}[\mu\mathbf{t}'. \oplus \{tc : \mathbf{t}', done : \mathbf{end}\}])$ and (*ii*) $(\mu\mathbf{t}'. \&\{tm : \mathbf{t}', over : \mathbf{end}\}, \mathcal{A}[\mathbf{end}])$. Observe that $T_G = \mathcal{A}[\mu\mathbf{t}'. \oplus \{tc : \mathbf{t}', done : \mathbf{end}\}]$. Hence, we have $T'_G \leq T_G$ with

$$\mathcal{R} = \{(T'_G, T_G), (\mathbf{end}, \mathbf{end}), (\mu\mathbf{t}'.\&\{tm : \mathbf{t}', over : \mathbf{end}\}, \mu\mathbf{t}.\&\{tm : \mathbf{t}, over : \mathbf{end}\})\}$$

and $\mathcal{R}$ is a controllable subtyping relation.

We show that fair asynchronous subtyping is sound w.r.t. fair refinement. In fact, fair asynchronous subtyping can be seen as a sound coinductive characterisation of fair refinement. Namely this result gives an operational justification to the syntactical definition of fair asynchronous session subtyping. Note that $\leq$ is not complete w.r.t. $\sqsubseteq$, see Example 7.

**Theorem 3.** *Given two session types $T$ and $S$, if $T \leq S$ then $T \sqsubseteq S$.*

*Example 7.* Let $T = \oplus\{l_1 : \&\{l_3 : \mathbf{end}\}\}$ and $S = \&\{l_3 : \oplus\{l_1 : \mathbf{end},\ l_2 : \mathbf{end}\}\}$. We have $T \sqsubseteq S$, but $T$ is not a fair asynchronous subtype of $S$ since $\{l_1\} \neq \{l_1, l_2\}$, i.e., covariance of outputs is not allowed.

Unfortunately, fair asynchronous session subtyping is also undecidable. The proof is similar to the one of undecidability of fair refinement, in particular we proceed by reduction from the termination problem in queue machines.

**Theorem 4.** *Given two session types $T$ and $S$, it is in general undecidable to check whether $T \leq S$.*

## 4 A Sound Algorithm for Fair Asynchronous Subtyping

We propose an algorithm which soundly verifies whether a session type is a fair asynchronous subtype of another. The algorithm relies on building a tree whose nodes are labelled by configurations of the simulation game induced by Definition 12. The algorithm analyses the tree to identify *witness* subtrees which contain input contexts that are growing following a recognisable pattern.

*Example 8.* Recall the satellite communication example (Figure 1). The spacecraft with protocol $T_S$ may be a replacement for an older generation of spacecraft which follows the more complicated protocol $T'_S$, see Figure 2. Type $T'_S$ notably allows the reception of telecommands to be interleaved with the emission of telemetries. The new spacecraft may safely replace the old one because $T_S \leq T'_S$.

However, checking $T_S \leq T'_S$ leads to an infinite accumulation of input contexts, hence it requires to consider infinitely many pairs of session types. E.g., after $T_S$ selects the output label *tm* twice, the subtyping simulation game considers the pair $(T_S, T''_S)$, where also $T''_S$ is in Figure 2. The pairs generated for this example illustrate a common recognisable pattern where some branches grow infinitely (the *tc*-branch), while others stay stable throughout the derivation (the *done*-branch). The crux of our algorithm is to use a finite parametric characterisation of the infinitely many pairs occurring in the check of $T_S \leq T'_S$.

The *simulation tree* for $T \leq S$, written $simtree(T, S)$, is the labelled tree representing the simulation game for $T \leq S$, i.e., $simtree(T, S)$ is a tuple $(N, n_0, \twoheadrightarrow, \lambda)$ where $N$ is its set of nodes, $n_0 \in N$ is its root, $\twoheadrightarrow$ is its transition function, and $\lambda$ is its labelling function, such that $\lambda(n_0) = (S, T)$. We omit the formal definition of $\twoheadrightarrow$, as it is straightforward from Definition 12 following the subtyping simulation game discussed after that definition. We give an example below.

Notice that the simulation tree $simtree(T, S)$ is defined only when $S$ is controllable, since $T \leq S$ holds without needing to play the subtyping simulation game if $S$ is not controllable. We say that a branch of $simtree(T, S)$ is *successful* if it is infinite or if it finishes in a leaf labelled by $(\mathbf{end}, \mathbf{end})$. All other branches are *unsuccessful.* Under the assumption that $S$ is controllable, we have that all branches of $simtree(T, S)$ are successful if and only if $T \leq S$. As a consequence checking whether all branches of $simtree(T, S)$ are successful is generally undecidable. It is possible to identify a branch as successful if it visits finitely many pairs (or node labels), see Example 6; but in general a branch may generate infinitely many pairs, see Examples 8 and 12.

In order to support types that generate unbounded accumulation, we characterise finite subtrees — called witness subtrees, see Definition 13 — such that all the branches that traverse these finite subtrees are guaranteed to be successful.

*Notation* We give a few auxiliary definitions and notations. Hereafter $\mathcal{A}$ and $\mathcal{A}'$ range over *extended* input contexts, i.e., input contexts that may contain distinct holes with the same index. These are needed to deal with unfoldings of input contexts, see Example 9.

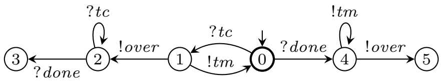

$$T'_S = \mu\mathbf{t}\,.\&\{\ tc :\ \ \oplus\{tm : \mathbf{t}, over : \mu\mathbf{t}'.\ \&\{tc : \mathbf{t}', done : \mathbf{end}\}\},$$
$$done : \mu\mathbf{t}''. \oplus \{tm : \mathbf{t}'', over : \mathbf{end}\}\}$$
$$T''_S = \ \ \&\{\ tc :\ \ \&\{\ tc :\ \ T'_S,$$
$$done : \mu\mathbf{t}''. \oplus \{tm : \mathbf{t}'', over : \mathbf{end}\}\ \},$$
$$done : \mu\mathbf{t}''. \oplus \{tm : \mathbf{t}'', over : \mathbf{end}\}\ \ \ \}$$

**Fig. 2.** $T'_S$ is an alternative session type for $T_S$, see Example 8.

The set of *reductions* of an input context $\mathcal{A}$ is the minimal set $\mathcal{S}$ s.t. $(i)$ $\mathcal{A} \in \mathcal{S}$; $(ii)$ if $\&\{l_i : \mathcal{A}_i\}_{i\in I} \in \mathcal{S}$ then $\forall i \in I.\mathcal{A}_i \in \mathcal{S}$ and $(iii)$ if $\mu\mathbf{t}.\mathcal{A}' \in \mathcal{S}$ then $\mathcal{A}'\{^{\mu\mathbf{t}.\mathcal{A}'}/_{\mathbf{t}}\} \in \mathcal{S}$. Notice that due to unfolding (item $(iii)$), the reductions of an input context may contain extended input contexts. Moreover, given a reduction $\mathcal{A}'$ of $\mathcal{A}$, we have that $holes(\mathcal{A}') \subseteq holes(\mathcal{A})$.

*Example 9.* Consider the following extended input contexts:

$$\mathcal{A}_1 = \mu\mathbf{t}.\ \&\{l_1 : [\,]^1,\ l_2 : \&\{l_3 : \mathbf{t}\}\} \qquad \mathcal{A}_2 = \&\{l_3 : \mu\mathbf{t}.\ \&\{l_1 : [\,]^1,\ l_2 : \&\{l_3 : \mathbf{t}\}\}\}$$

$$\mathsf{unfold}(\mathcal{A}_1) = \&\{l_1 : [\,]^1,\ l_2 : \&\{l_3 : \mu\mathbf{t}.\ \&\{l_1 : [\,]^1,\ l_2 : \&\{l_3 : \mathbf{t}\}\}\}\}$$

Context $\mathcal{A}_2$ is a reduction of $\mathcal{A}_1$, i.e., one can reach $\mathcal{A}_2$ from $\mathcal{A}_1$, by unfolding $\mathcal{A}_1$ and executing the input $l_2$. Context $\mathsf{unfold}(\mathcal{A}_1)$ is also a reduction of $\mathcal{A}_1$. Observe that $\mathsf{unfold}(\mathcal{A}_1)$ contains two distinct holes indexed by 1.

Given an extended context $\mathcal{A}$ and a set of hole indices $K$ such that $K \subseteq holes(\mathcal{A})$, we use the following shorthands. Given a type $T_k$ for each $k \in K$, we write $\mathcal{A}\lfloor T_k \rfloor^{k\in K}$ for the extended context obtained by replacing each hole $k \in K$ in $\mathcal{A}$ by $T_k$. Also, given an extended context $\mathcal{A}'$ we write $\mathcal{A}\langle \mathcal{A}' \rangle^K$ for the extended context obtained by replacing each hole $k \in K$ in $\mathcal{A}$ by $\mathcal{A}'$. When $K = \{k\}$, we often omit $K$ and write, e.g., $\mathcal{A}\langle \mathcal{A}' \rangle^k$ and $\mathcal{A}\lfloor T_k \rfloor^k$.

*Example 10.* Using the above notation and posing $\mathcal{A} = \&\{tc : [\,]^1, done : [\,]^2\}$, we can rewrite $T''_S$ (Figure 2) as $\mathcal{A}\langle \mathcal{A}\lfloor T'_S \rfloor^1 \rangle^1 \lfloor \mu\mathbf{t}''. \oplus \{tm : \mathbf{t}'', over : \mathbf{end}\} \rfloor^2$.

*Example 11.* Consider the session type below

$$S = \&\{l_1 : \&\{l_1 : T_1,\ l_2 : T_2,\ l_3 : T_3\},\ l_2 : \&\{l_1 : T_1,\ l_2 : T_2,\ l_3 : T_3\},\ l_3 : T_3\}.$$

Posing $\mathcal{A} = \&\{l_1 : [\,]^1, l_2 : [\,]^2, l_3 : [\,]^3\}$ we have $holes(\mathcal{A}) = \{1, 2, 3\}$. Assuming $J = \{1, 2\}$ and $K = \{3\}$, we can rewrite $S$ as $\mathcal{A}\langle \mathcal{A}\lfloor T_j \rfloor^{j\in J} \rangle^J \lfloor T_k \rfloor^{k\in K}$.

*Example 12.* Figure 3 shows the partial simulation tree for $T_S \leq T'_S$, from Figures 1 and 2 (ignore the dashed edges for now). Notice how the branch leading to the top part of the tree visits only finitely many node labels (see dotted box), however the bottom part of the tree generates infinitely many labels, see the path along the $!tm$ transitions in the dashed box.

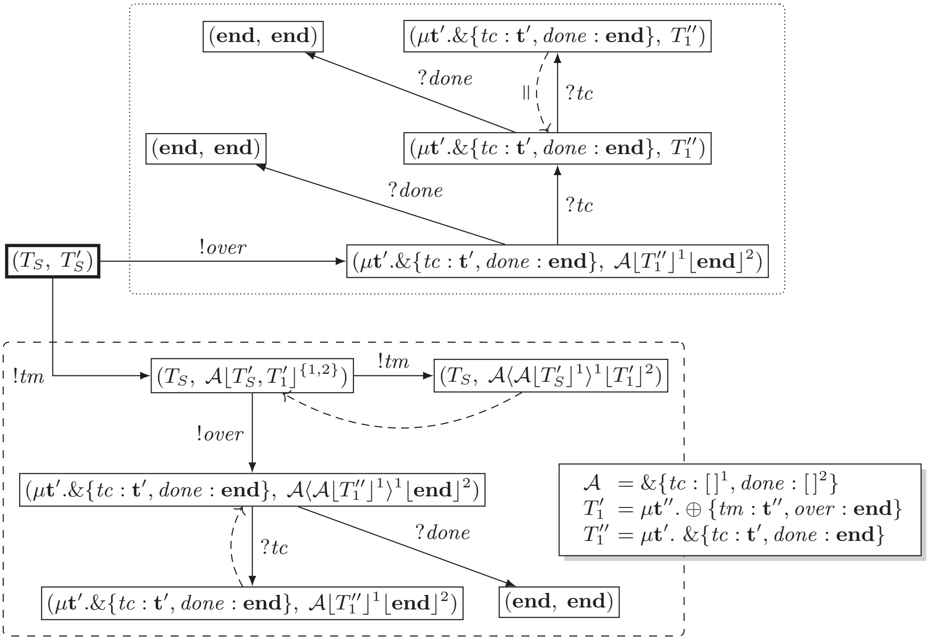

**Fig. 3.** Simulation tree for $T_S \leq T'_S$ (Figures 1 and 2), the root of the tree is in bold.

*Witness subtrees* Next, we define witness trees which are finite subtrees of a simulation tree which we prove to be successful. The role of the witness subtree is to identify branches that satisfy a certain accumulation pattern. It detects an input context $\mathcal{A}$ whose holes fall in two categories: $(i)$ growing holes (indexed by indices in $J$ below) which lead to an infinite growth and $(ii)$ constant holes (indexed by indices in $K$ below) which stay stable throughout the simulation game. The definition of witness trees relies on the notion of *ancestor* of a node $n$, which is a node $n'$ (different from $n$) on the path from the root $n_0$ to $n$. We illustrate witness trees with Figure 3 and Example 13.

**Definition 13 (Witness Tree).** *A tree* $(N, n_0, \twoheadrightarrow, \lambda)$ *is a* witness tree *for* $\mathcal{A}$*, such that* $holes(\mathcal{A}) = I$*, with* $\emptyset \subseteq K \subset I$ *and* $J = I \setminus K$*, if all the following conditions are satisfied:*

1. *for all* $n \in N$ *either* $\lambda(n) = (T, \mathcal{A}'\langle \mathcal{A}\lfloor S_j \rfloor^{j \in J} \rangle^J \lfloor S_k \rfloor^{k \in K})$ *or* $\lambda(n) = (T, \mathcal{A}'\langle \mathcal{A}\langle \mathcal{A}\lfloor S_j \rfloor^{j \in J} \rangle^J \rangle^J \lfloor S_k \rfloor^{k \in K})$*, where* $\mathcal{A}'$ *is a reduction of* $\mathcal{A}$*, and it holds that*
   - $holes(\mathcal{A}') \subseteq K$ *implies that* $n$ *is a leaf and*
   - *if* $\lambda(n) = (T, \mathcal{A}[S_i]^{i \in I})$ *and* $n$ *is not a leaf then* $\mathsf{unfold}(T)$ *starts with an output selection;*
2. *each leaf* $n$ *of the tree satisfies one of the following conditions:*
   *(a)* $\lambda(n) = (T, S)$ *and* $n$ *has an ancestor* $n'$ *s.t.* $\lambda(n') = (T, S)$

*(b)* $\lambda(n) = (T, \mathcal{A}\langle\mathcal{A}\lfloor S_j\rfloor^{j\in J}\rangle^J\lfloor S_k\rfloor^{k\in K})$ *and n has an ancestor* $n'$ *s.t.* $\lambda(n') = (T, \mathcal{A}[S_i]^{i\in I})$

*(c)* $\lambda(n) = (T, \mathcal{A}[S_i]^{i\in I})$ *and*
*n has an ancestor* $n'$ *s.t.* $\lambda(n') = (T, \mathcal{A}\langle\mathcal{A}\lfloor S_j\rfloor^{j\in J}\rangle^J\lfloor S_k\rfloor^{k\in K})$

*(d)* $\lambda(n) = (T, \mathcal{A}'[S_k]^{k\in K'})$ *where* $K' \subseteq K$

*and for all leaves* $(T, S)$ *of type* (2c) *or* (2d) $T \leq S$ *holds.*

Intuitively Condition (1) says that a witness subtree consists of nodes that are labelled by pairs $(T, S)$ where $S$ contains a fixed context $\mathcal{A}$ (or a reduction/repetition thereof) whose holes are partitioned in growing holes $(J)$ and constant holes $(K)$. Whenever all growing holes have been removed from a pair (by reduction of the context) then this means that the pair is labelling a leaf of the tree. In addition, if the initial input is limited to only one instance of $\mathcal{A}$, the l.h.s. type starts with an output selection so that this input cannot be consumed in the subtyping simulation game.

Condition 2 says that all leaves of the tree must validate certain conditions from which we can infer that their continuations in the full simulation tree lead to successful branches. Leaves satisfying Condition (2a) straightforwardly lead to successful branches as the subtyping simulation game, starting from the corresponding pair, has been already checked starting from its ancestor having the same label. Leaves satisfying Condition (2b) lead to an infinite but regular "increase" of the types in $J$-indexed holes — following the same pattern of accumulation from their ancestor. The next two kinds of leaves must additionally satisfy the subtyping relation — using witness trees inductively or based on the fact they generate finitely many labels. Leaves satisfying Condition (2c) lead to regular "decrease" of the types in $J$-indexed holes — following the same pattern of reduction from their ancestor. Leaves satisfying Condition (2d) use only constant $K$-indexed holes because, by reduction of the context $\mathcal{A}'$, the growing holes containing the accumulation $\mathcal{A}$ have been removed.

*Remark 1.* Definition 13 is parameterised by an input context $\mathcal{A}$. We explain how such contexts can be identified while building a simulation tree in Section 5.

*Example 13.* In the tree of Figure 3 we highlight two subtrees. The subtree in the dotted box is not a witness subtree because it does not validate Condition (1) of Definition 13, i.e., there is an intermediary node with a label in which the r.h.s type does not contain $\mathcal{A}$.

The subtree in the dashed box is a witness subtree with 3 leaves, where the dashed edges represent the ancestor relation, $\mathcal{A} = \&\{tc : [\,]^1, done : [\,]^2\}$, $J = \{1\}$ and $K = \{2\}$. We comment on the leaves clockwise, starting from $(\mathbf{end}, \mathbf{end})$, which satisfies Condition (2d). The next leaf satisfies condition (2c), while the final leaf satisfies Condition (2b).

*Algorithm* Given two session types $T$ and $S$ we first check whether $S$ is uncontrollable. If this is the case we immediately conclude that $T \leq S$. Otherwise, we proceed in four steps.

**S1** We compute a finite fragment of $\mathit{simtree}(T, S)$, stopping whenever $(i)$ we encounter a leaf (successful or not), $(ii)$ we encounter a node that has an ancestor as defined in Definition 13 (Conditions (2a), (2b), and (2c)), $(iii)$ or the length of the path from the root of $\mathit{simtree}(T, S)$ to the current node exceeds a bound set to two times the depth of the AST of $S$. This bound allows the algorithm to explore paths that will traverse the super-type at least twice. We have empirically confirmed that it is sufficient for all examples mentioned in Section 5.
**S2** We remove subtrees from the tree produced in **S1** corresponding to successful branches of the simulation game which contain finitely many labels. Concretely, we remove each subtree whose each leaf $n$ is either successful or has an ancestor $n'$ such that $n'$ is in the same subtree and $\lambda(n) = \lambda(n')$.
**S3** We extract subtrees from the tree produced in **S2** that are potential *candidates* to be subsequently checked. The extraction of these finite candidate subtrees is done by identifying the forest of subtrees rooted in ancestor nodes which do not have ancestors themselves.
**S4** We check that each of the candidate subtrees from **S3** is a witness tree.

If an unsuccessful leaf is found in **S1**, then the considered session types are not related. In **S1**, if the generation of the subtree reached the bound before reaching an ancestor or a leaf, then the algorithm is unable to give a decisive verdict, i.e., the result is *unknown*. Otherwise, if all checks in **S4** succeed then the session types are in the fair asynchronous subtyping relation. In all other cases, the result is *unknown* because a candidate subtree is not a witness.

*Example 14.* We illustrate the algorithm above with the tree in Figure 3. After **S1**, we obtain the whole tree in the figure (11 nodes). After **S2**, all nodes in the dotted boxed are removed. After **S3** we obtain the (unique) candidate subtree contained in the dashed box. This subtree is identified as a witness subtree in **S4**, hence we have $T_S \leq T'_S$.

We state the main theorem that establishes the soundness of our algorithm, where $\twoheadrightarrow^*$ is the reflexive and transitive closure of $\twoheadrightarrow$.

**Theorem 5.** *Let $T$ and $S$ be session types s.t. $\mathit{simtree}(T, S) = (N, n_0, \twoheadrightarrow, \lambda)$. If $\mathit{simtree}(T, S)$ contains a witness subtree with root $n$ then for every node $n' \in N$ s.t. $n \twoheadrightarrow^* n'$, either $n'$ is a successful leaf, or there exists $n''$ s.t. $n' \twoheadrightarrow n''$.*

We can conclude that if the candidate subtrees of $\mathit{simtree}(T, S)$ identified with the strategy explained above are also witness subtrees, then we have $T \leq S$.

## 5 Implementation

To evaluate our algorithm, we have produced a Haskell implementation of it, which is available on GitHub [31]. Our tool takes two session types $T$ and $S$ as input then applies Steps **S1** to **S4** to check whether $T \leq S$. A user-provided bound can be given as an optional argument. We have run our tool on a dozen of examples handcrafted to test the limits of our algorithm (inc. the examples discussed in this paper), as well as on the 174 tests taken from [6]. All of these tests terminate under a second.

For debugging and illustration purposes, the tool can optionally generate graphical representations of the simulation and witness trees, and check whether the given types are controllable. We give examples of these in [9].

Our tool internally uses automata to represent session types and uses strong bisimilarity instead of syntactic equality between session types. Using automata internally helps us identify candidate input contexts as we can keep track of states that correspond to the input context computed when applying Case (4) of Definition 12. In particular, we augment each local state in the automata representation of the candidate supertype with two counters: the $c$-counter keeps track of how many times a state has been used in an input $c$ontext; the $h$-counter keeps track of how many times a state has occurred within a $h$ole of an input context. We illustrate this with Figure 4 which illustrates the internal data structures our tool manipulates when checking $T_S \leq T'_S$ from Figures 1 and 2. The state indices of the automata in Figure 4 correspond to the ones in Figure 1 ($2^{\text{nd}}$ column) and Figure 2 ($3^{\text{rd}}$ column).

The first row of Figure 4 represents the root of the simulation tree, where both session types are in their respective initial state and no transition has been executed. We use state labels of the form $n_{c,h}$ where $n$ is the original identity of the state, $c$ is the value of the $c$-counter, and $h$ is the value of the $h$-counter. The second row depicts the configuration after firing transition $!tm$, via Case (4) of Definition 12. While the candidate subtype remains in state 0 (due to a self-loop) the candidate supertype is unfolded with $\mathsf{selUnfold}(T'_S)$ (Definition 10). The resulting automaton contains an additional state and two transitions. All previously existing states have their $h$-counter incremented, while the new state has its $c$-counter incremented. The third row of the figure shows the configuration after firing transition $!over$, using Case (4) of Definition 12 again. In this step, another copy of state 0 is added. Its $c$-counter is set to 2 since this state has been used in a context twice; and the $h$-counters of all other states are incremented.

Using this representation, we construct a candidate input context by building a tree whose root is a state $q_{c,h}$ such that $c > 1$. The nodes of the tree are taken from the states reachable from $q_{c,h}$, stopping when a state $q'_{c',h'}$ such that $c' < c$ is found. A leaf $q'_{c',h'}$ becomes a hole of the input context. The hole is a constant ($K$) hole when $h' = c$, and growing ($J$) otherwise. Given this strategy and the configurations in Figure 4, we successfully identify the context $\mathcal{A} = \&\{tc : [\,]^1, done : [\,]^2\}$ with $J = \{1\}$ and $K = \{2\}$.

## 6 Related and Future Work

*Related work* We first compare with previous work on refinement for asynchronous communication by some of the authors of this paper. The work in [10] also considers fair compliance, however here we consider binary (instead of multiparty) communication and we use a unique input queue for all incoming messages instead of distinct named input channels. Moreover, here we provide a

| Last transition | State of $T_S$ | Representation of $T'_S$ |
|---|---|---|
| $\epsilon$ | 0 |   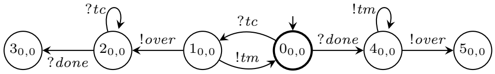 |
| $!tm$ | 0 |   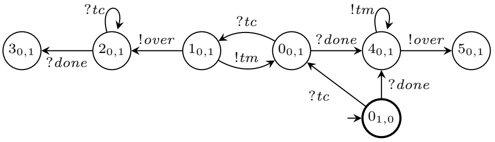 |
| $!over$ | 1 |   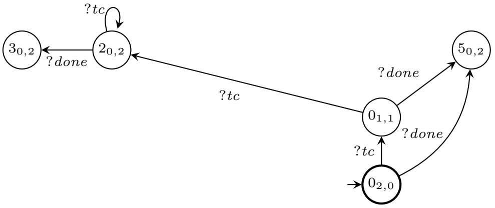 |

**Fig. 4.** Internal representation of the simulation tree for $T_S \leq T'_S$ (fragment).

sound characterisation of fair refinement using coinductive subtyping and provide a sound algorithm and its implementation. In [13] the asynchronous subtyping of [7, 14, 15, 26] is used to characterise refinement for a notion of correct composition based on the impossibility to reach a deadlock, instead of the possibility to reach a final successful configuration as done in the present paper. The refinement from [13] does not support examples such as those in Figure 1.

Concerning previous notions of synchronous subtyping, Gay and Hole [17,18] first introduced the notion of subtyping for *synchronous* session types, which is decidable in quadratic time [22]. This subtyping only supports covariance of outputs and contravariance of inputs, but does not address anticipation of outputs. Padovani studied a notion of fair subtyping for *synchronous* multi-party session types in [29]. This work notably considers the notion of *viability* which corresponds, in the synchronous multiparty setting, to our notion of controllability. We use the term controllability instead of viability following the tradition of service contract theories like those based on Petri nets [25, 33] or process calculi [12]. In contrast to [29], asynchronous communication makes it much more involved to characterise controllability in a decidable way, as we do in this paper. Fair refinement in [29] is characterised by defining a coinductive relation on normal form of types, obtained by removing inputs leading to uncontrollable continuations. Instead of using normal forms, we remove these inputs during the asynchronous subtyping check. A limited form of variance on output is also admitted in [29]. Covariance between the outputs of a subtype and those of a supertype is possible when the additional branches in the supertype are not needed to have compliance with potential partners. In [29] this check is made possible by exploiting a *difference* operation [29, Definition 3.15] on types, which synthesises a new type representing branches of one type that are absent in the other. We observe that the same approach cannot work to introduce variance on outputs in an asynchronous setting. Indeed the interplay between output anticipation and recursion could generate differences in the branches of a subtype and a supertype that cannot be statically represented by a (finite) session type.

Padovani also studied an alternative notion of fair *synchronous* subtyping in [28]. Although the contribution of that paper refers to session types, the formal framework therein seems to deviate from the usual session type approach. In particular, it considers shared channel communication instead of binary channels: when a partner emits a message, it is possible to have a race among several potential receivers for consuming it. As a consequence of this alternative semantics, the subtyping in [28] does not admit variance on input. Another difference with respect to session type literature is the notion of *success* among interacting sessions: a composition of session is successful if at least one participant reaches an internal successful state. This approach has commonalities with testing [27], where only the test composed with the system under test is expected to succeed, but differs from the typical notion of success considered for session types. In [2,3] (resp. [14]) it was proved that the Gay-Hole synchronous session subtyping (resp. orphan message free asynchronous subtyping) coincides with refinement induced by a successful termination notion requiring interacting processes to be *both* in the **end** state (with empty buffers, in the asynchronous case).

Several variants of asynchronous session subtyping have been proposed in [14, 15, 26] and further studied in our earlier work [6, 7, 13]. All these variants have been shown to be undecidable [7, 8, 23]. Moreover, all these subtyping relations are (implicitly) based on an unfair notion of compliance. Concretely, the definition of asynchronous subtyping introduced in this paper differs from the one in [14,15] since no additional constraint guaranteeing absence of orphan-messages is considered. Such a constraint requires the subtype not to have output loops whenever an output anticipation is performed, thus guaranteeing that at least one input is performed in all possible paths. In this paper, absence of orphan messages is guaranteed by enforcing types to (fairly) reach a successful termination. Moreover, our novel subtyping differs from those in [14, 15, 26] since we use recursive input contexts (and not just finite ones) for the first time — this is necessary to obtain $T'_G \leq T_G$ and $T_S \leq T'_S$ (see Figures 1 and 2). Notice that not imposing the above mentioned orphan-message-free constraint of [14, 15] is consistent with recursive input contexts that allows for input loops in the supertype whenever an output anticipation is performed. In [6], we proposed a sound algorithm for the asynchronous subtyping in [14]. The sound algorithm that we present in this paper substantially differs from that of [6]. Here we use witness trees that take under consideration both increasing and decreasing of accumulated input. In [6], instead, only regular growing accumulation is considered.

*Future work* In future work, we will investigate how to support output variance in fair asynchronous subtyping. We also plan to study fairness in the context of asynchronous multiparty session types, as fair compliance and refinement extend naturally to several partners. Finally, we will investigate a more refined termination condition for our algorithm using ideas from [6, Definition 11].
